# Ultralow-power switching via defect engineering in germanium telluride phase-change memory devices

**DOI:** 10.1038/ncomms10482

**Published:** 2016-01-25

**Authors:** Pavan Nukala, Chia-Chun Lin, Russell Composto, Ritesh Agarwal

**Affiliations:** 1 Department of Materials Science and Engineering, University of Pennsylvania, Philadelphia, Pennsylvania 19104, USA

## Abstract

Crystal–amorphous transformation achieved via the melt-quench pathway in phase-change memory involves fundamentally inefficient energy conversion events; and this translates to large switching current densities, responsible for chemical segregation and device degradation. Alternatively, introducing defects in the crystalline phase can engineer carrier localization effects enhancing carrier–lattice coupling; and this can efficiently extract work required to introduce bond distortions necessary for amorphization from input electrical energy. Here, by pre-inducing extended defects and thus carrier localization effects in crystalline GeTe via high-energy ion irradiation, we show tremendous improvement in amorphization current densities (0.13–0.6 MA cm^−2^) compared with the melt-quench strategy (∼50 MA cm^−2^). We show scaling behaviour and good reversibility on these devices, and explore several intermediate resistance states that are accessible during both amorphization and recrystallization pathways. Existence of multiple resistance states, along with ultralow-power switching and scaling capabilities, makes this approach promising in context of low-power memory and neuromorphic computation.

## Abstract (web summary)

Phase change memories involve crystalline-to-amorphous transformations which require high current densities. Here, the authors introduce extended defects in GeTe crystals, reduce the current densities necessary for amorphization and obtain low-power, scalable memories with multiple resistance states.

## Graphical abstract

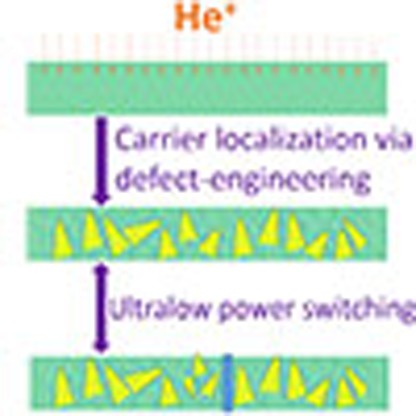

Phase-change materials (PCMs), which rapidly and reversibly transform from crystalline to amorphous phase, are viable alternatives to the relatively slow non-volatile flash memory technology^1^ with random access capability. One of the problems with PCMs is the use of high programming currents (and current densities) during the crystal–amorphous transformation (RESET) achieved conventionally via the melt-quench pathway^2^; and reducing the active device volumes has been pursued as a potential solution to mitigate this problem^3^,^4^,^5^,^6^,^7^,^8^. While reports on phase-change line and bridge devices demonstrated lesser RESET currents by shrinking the volume of the PCM directly^4^,^5^,^8^, works on PCM devices with carbon nanotube electrodes^6^,^7^ showed very low RESET currents (∼5 μA) by minimizing the contact areas and hence the active device volumes (35 × 3 × 3 nm)^6^. Alternative approaches to reducing the RESET currents include lowering the melting point of the PCM by doping them with nitrogen or silicon^9^,^10^. Although these approaches illustrate intelligent device designs based on geometry and chemical doping, none of them have been able to reduce the high amorphization current densities (∼50 MA cm^−2^)^7^ that are responsible for device degradation issues owing to chemical segregation and heat^11^, thus precluding the widespread commercialization of PCM technology.

In the melt-quench pathway, work required to amorphize a crystal is extracted from the heat generated by input electrical energy via inelastic carrier-phonon scattering; and this involves several fundamentally inefficient energy conversion events^12^ manifested as large writing current densities. Engineering carrier localization effects by pre-inducing extended defects in crystalline PCM enhances carrier–lattice coupling^13^, enabling efficient exchange of energy back and forth between the carriers and the lattice^14^,^15^. The input electrical energy, hence, can directly perform work on the lattice with minimal loss in the form of heat (wasteful energy) and introduce critical bond distortions required for amorphization^16^, potentially lowering the writing current densities.

Here we select crystalline GeTe (*R3m*), a simple binary PCM system, to demonstrate drastic improvement in amorphization current (and power) densities achieved by introducing carrier localization near the Fermi level (*E*_F_). All known phases of crystalline GeTe show *p*-type metallic conduction owing to the presence of large concentration (order of 10^20^ cm^−3^) of intrinsic Ge vacancies (*p*-type dopants)^17^. The carriers near the Fermi level (*E*_F_), which participate in transport, are delocalized and couple weakly with the lattice. By pre-inducing extended defects using high-energy He^+^ ion irradiation, we show for GeTe devices in the crystalline phase that the carriers at *E*_F_ can be localized, and hence strongly couple with the lattice^14^,^15^. These devices transformed to an amorphous phase via the defect-based pathway^13^,^18^, at current densities (*j*_s_) of 0.13–0.5 MA cm^−2^ significantly lower than *j*_s_=50 MA cm^−2^ observed in the melt-quench pathway^6^,^7^. Furthermore, we illustrate scaling of switching currents with device volumes, and reversible and repeatable low-power switching from defect-engineered crystalline states to amorphous phase.

## Results

### Inducing carrier localization in single-crystalline GeTe nanowires

**Figure 1:**
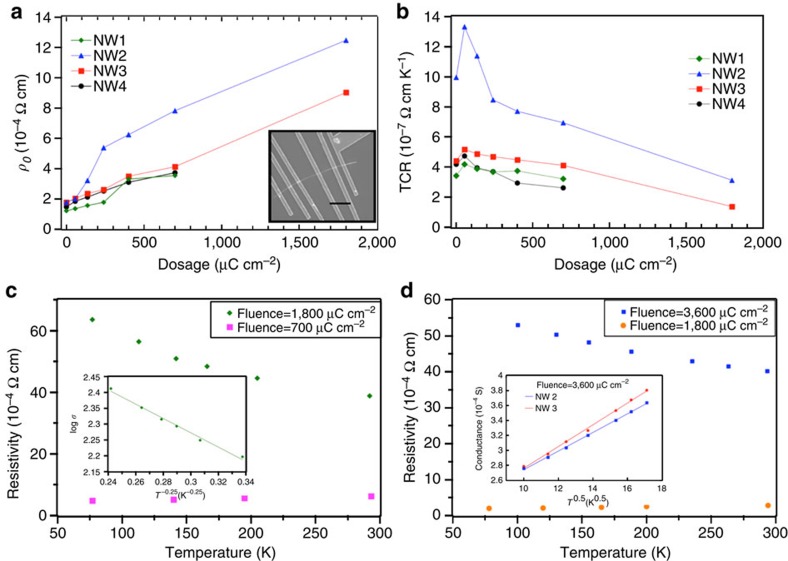
Transport measurements on crystalline GeTe nanowires irradiated at various dosages. (**a**) Saturation resistivity (*ρ*_0_) plots as a function of dosage on four representative nanowires (NW 1–4), showing an increase in *ρ*_0_ with dosage, in the metallic state. Inset: Scanning electron microscope image of NW 1, a representative multiple probe nanowire devices for transport measurements. Scale bar, 2 μm. (**b**) Temperature coefficient of resistivity (TCR) plots as a function of dosage on four representative nanowires (NW 1, 2, 3 and 4), showing an initial increase in TCR followed by a subsequent decrease with dosage in the metallic state. (**c**) Temperature–resistance plots for NW 1 at dosages 700 μC cm^−2^ (magenta) and 1,800 μC cm^−2^ (green), signifying a metal–insulator transition. Inset: variable range hopping (VRH) conduction behaviour observed at 1,800 μC cm^−2^, dosage confirming the insulating state. (**d**) Temperature–resistance plots for NW 2 at 1,800 μC cm^−2^ (orange) and 3,600 μC cm^−2^ (blue), signifying a metal–dirty metal transition. Inset: power law conduction behaviour observed for NWs 2 and 3 at 3,600 μC cm^−2^ confirming dirty metallic nature.

Single-crystalline GeTe nanowires were synthesized using the vapour–liquid–solid mechanism^19^, and multiple electrode devices were fabricated using electron-beam lithography, and encapsulated with 30 nm of SiO_2_ (Fig. 1a, inset; Methods)^13^. All devices were irradiated using 2 MeV He^+^ ions, in a Rutherford backscattering set-up, at different dosages, and beam currents not exceeding 30 nA (ref. 20; Methods; Supplementary Note 1). Our stopping and range of ions in matter calculations (Supplementary Note 1) showed that there is a 2% loss in the incident energy upon the penetration of He^+^ ions through our nanowires, ensuring extended defect formation via knock-on damage and dismissing any role of ion implantation (He bubble or void formation)^21^. To understand the effect of pre-induced defects on the transport characteristics in nanowire devices, we performed temperature-dependent resistivity measurements after ion-beam exposure at various dosages. Resistivity was evaluated as *ρ=R*_NW_*A/l*_d_, where *R*_NW_ is the resistance of the nanowire obtained by subtracting the contact resistance measured in a multiple probe configuration (Fig. 1a, inset) from the total device resistance. *l*_d_ and *A* are the length and cross-sectional area of the nanowire device, respectively (see Supplementary Note 2, raw data shown in Supplementary Fig. 1). At dosages up to 700 μC cm^−2^, the resistivity of all our devices increased linearly with increasing temperature above 30 K and exhibited a saturation value (*ρ*_*0*_) below 30 K. This behaviour is typical of metals, where dominant contribution to resistivity at low temperatures is from temperature-independent carrier-defect scattering and carrier-phonon scattering at high temperatures^13^. *ρ*_0_ depends on the defect density, and conversely can be used as a measurable metric for the same in the metallic state. As illustrated for representative devices (labelled nanowires 1–4) in Fig. 1a, *ρ*_0_ increased with increasing dosage (from 0 to 700 μC cm^−2^), consistent with increasing pre-induced defect density in the material.

Another quantity that is sensitive to the defect concentration in metallic state is the slope of temperature–resistivity plots in the linear increase regime (temperature coefficient of resistivity, TCR), which decreases with increasing defect density^22^,^23^, nevertheless remaining positive. Positive TCR is a characteristic of transport via delocalized carriers in a metal, which can be described by Boltzmann transport equation^24^. In all our devices (representative devices nanowires 1–4 shown in Fig. 1b), although TCR showed an initial increase with dosage up to 50 μC cm^−2^ (for reasons see Supplementary Note 3 and Supplementary Fig. 2), subsequently up to 700 μC cm^−2^ it showed a decreasing trend to a less positive value, consistent with increasing defect density. More importantly, TCR remained positive, suggesting that at these low dosages (<700 μC cm^−2^) carrier localization effects are insignificant.

At higher dosages (1,800–3,600 μC cm^−2^), however, the resistivity of all the tested devices showed a non-linear decrease with increasing temperature (TCR is negative but cannot be uniquely defined), which is a signature of localized carriers at *E*_F_ participating in transport^25^. This demonstrates an electronic transformation of crystalline GeTe from a metallic state to dirty metallic (*ρ*_0_ is finite) or insulating states (*ρ*_0_→∞)^15^. The exact dosage at which this transformation occurred varied from device to device, but all devices showed localization effects in transport above a dosage of 3,600 μC cm^−2^. For instance, the device nanowire 1 (Fig. 1c) transformed to an insulating state demonstrating variable range hopping (VRH) conduction (Fig. 1c, inset) at a dosage of 1,800 μC cm^−2^. Devices nanowires 2 and 3 (Fig. 1d) transformed to dirty metal state at 3,600 μC cm^−2^, demonstrating a power law conduction (*σ* varying as *T*^0.5^), a characteristic of disordered metals showing weak localization effects (Fig. 1d, inset)^25^. The stability of these defect-engineered states was tested by heating the devices to 200 °C and monitoring the change in resistance with time; and they showed no change in resistance for 36 h (Supplementary Fig. 3), suggesting that they are thermodynamically stable (Supplementary Note 4).

**Figure 2:**
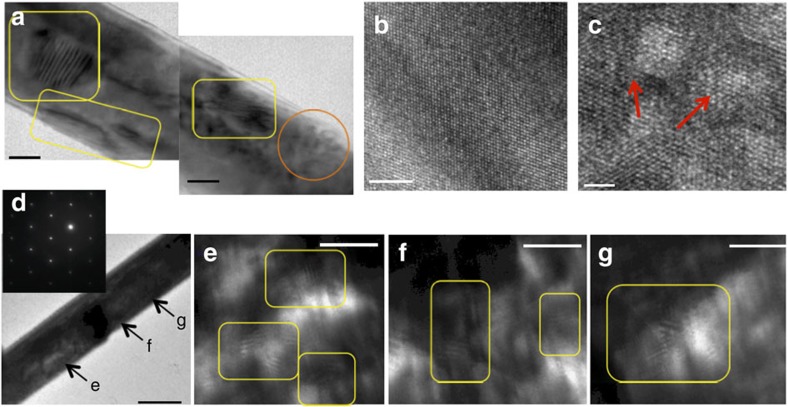
Structural analysis on crystalline GeTe nanowires irradiated at various dosages. (**a**) Bright-field TEM image showing stacking faults (yellow rectangles) and dislocation loops (orange circles) induced randomly in a nanowire irradiated with a dosage of 45 μC cm^−2^. Scale bar, 50 nm (**b**,**c**) High-resolution TEM images of a nanowire before (**b**) and after (**c**) ion irradiation with a dosage of 100 μC cm^−2^ showing defect tetrahedra (red arrows). Scale bar, 5 nm in **b**, and 2 nm in **c**. (**d**) Bright-field TEM image of a nanowire ion irradiated with large fluences (1,800 μC cm^−2^, inset). Selected area electron diffraction showing that the nanowire is still single crystalline with the satellite spots corresponding to the existence of defects. Scale bar, 200 nm. (**e**–**g**) Zoomed in, dark-field TEM images of different regions marked in **d**, all showing many intersecting defect templates, a structural feature that corresponds to electron localization. Scale bar, 20 nm (**e**,**f**,**g**).

To understand the structural nature of the radiation induced defects, we performed transmission electron microscopy (TEM) on nanowire devices assembled on TEM compatible platform^18^ and exposed to different dosages of He^+^ ion irradiation. At modest dosages (40–100 μC cm^2^), where the devices remain metallic, we observed the formation of dislocation loops, two-dimensional defects (stacking faults) and defect tetrahedra—formed due to vacancy/interstitial supersaturation following knockout of atoms (Fig. 2a–c)^26^. The spatial distribution of these defects along the nanowire is sporadic. For irradiation at higher dosages (1,800 μC cm^−2^), the entire nanowire became replete with intersecting two-dimensional defects (as illustrated in different regions of a representative nanowire in Fig. 2d–g), still being single crystalline (Fig. 2d, inset), and this stage corresponds to electronic states where carrier localization dominates transport (Fig. 1c,d). It is important to emphasize that our devices are capped with a conformal coating of SiO_2_ (Methods), which prevents these pre-induced defects from annihilating at the surface of the nanowires (the only possible sink) at high temperatures, contributing to their thermal stability (Supplementary Fig. 3). Furthermore, we did not see any evidence of He bubble trapping in GeTe, another common defect observed in materials irradiated with He^+^ ions^26^ (Supplementary Notes 1 and 5; Supplementary Fig. 4) owing to the high energy of the incident ion beam^21^.

### Amorphization behaviour of defect-engineered nanowire devices

To verify the idea that pre-inducing defects and engineering carrier localization effects is beneficial for power reduction for amorphization, we studied the switching (crystal–amorphous) and volume scaling properties of the devices as a function of radiation dosage. We amorphized our devices in crystalline phase exposed to different dosages of ion irradiation, by applying a train of voltage pulses (50 ns) of increasing amplitude, separated by 1 s (to allow complete thermalization between pulses), until resistance increased abruptly by at least two orders of magnitude (see Supplementary Note 6 and Supplementary Fig. 5 for pulse shapes, and dynamic currents measured from a pulse). The crystal–amorphous transformation in all these devices occurred via a defect-based mechanism, where heat shock from an electrical pulse first creates extended defects (full and partial dislocations)^13^,^18^. The pre-induced defects, along with the defects created by electrical pulses, migrate with the hole-wind force and accumulate at a region of local inhomogeneity creating a defect template with intersecting defects, along which amorphization takes place beyond a critical defect density^13^,^25^. To understand the size scaling of RESET currents (*i*_s_) in this defect-based mechanism, and the influence of pre-induced defects on them, we compared RESET current densities (*j*_s_) as a function of device length (*l*_d_) at various dosages (Fig. 2; Supplementary Fig. 6a). These plots encompass complete information on size (length and cross-sectional area) dependence of *i*_s_. At dosages up to 700 μC cm^−2^, where no carrier localization effects were present, we observed that *j*_s_ increased with increasing dosage for any particular device length (Supplementary Fig. 6a–d). This illustrates that pre-induced defects can be detrimental for *i*_s_ (*j*_s_), if they do not induce any significant localization effects in transport (see Supplementary Note 7 for analysis of switching behaviour at low dosages).

**Figure 3:**
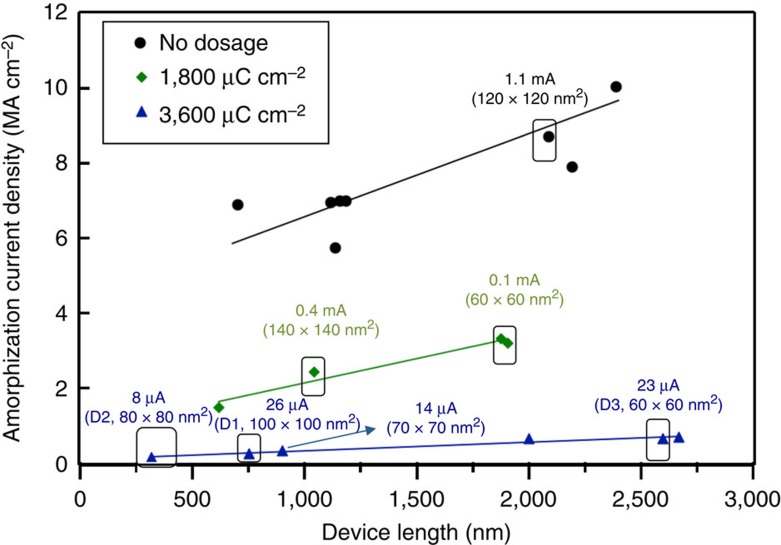
Scaling behaviour of switching properties of GeTe nanowire devices with pre-induced defects at different dosages. Plot showing amorphization current density (*j*_s_) as a function of device length for devices engineered into states where localized carriers dominate transport when nanowire devices were irradiated at very high dosages (1,800 and 3,600 μC cm^−2^). Upon comparison with non-irradiated devices (black data points), these devices showed a drastic reduction in switching current densities for amorphization, enabling very low-current switching for large devices. The switching currents (*i*_s_) and device cross-section dimensions are indicated on the plot, and the device volumes and comparison with devices switching via melt quench is shown in Supplementary Table 1. Three particular devices named D1, D2 and D3 defect engineered at a dosage of 3,600 μC cm^−2^ were selected for further analysis. Data shown in Figs 4, 5, 6 are on device D1.

However, at higher dosages (>1,800 μC cm^−2^), where the localization effects dominated transport (Fig. 1a–d), *j*_s_ (and *i*_s_) were drastically lowered (Fig. 3). At a dosage of 3,600 μC cm^−2^, *i*_s_ of the device (referred as D1) with active volume as large as 100 × 100 × 750 nm (see Supplementary Note 8) was as low as 26 μA (*j*_s_: 0.26 MA cm^−2^); and for a smaller device (80 × 80 × 320 nm, referred as D2), *i*_s_ was 8 μA (*j*_s_: 0.13 MA cm^−2^). The current densities and power densities for amorphizing these defect-engineered devices were ∼300 and 10^5^ times smaller, respectively, than those required by the melt-quench pathway^6^ (*i*_s_:5 μA, *j*_s_: 50 MA cm^−2^; Supplementary Note 8; Supplementary Table 1). With volume scaling also demonstrated on these devices, the absolute power required for switching very small volumes of active PCM^6^ is significantly lowered in this approach, and this can potentially mitigate issues such as thermal cross-talk and chemical segregation^11^. Furthermore, the importance of carrier localization and the role of lattice–carrier coupling in efficiently converting input electrical energy into the work required for amorphization resulting in significant lowering of switching powers are highlighted by these results.

### Recrystallization behaviour of defect-engineered nanowire devices

**Figure 4:**
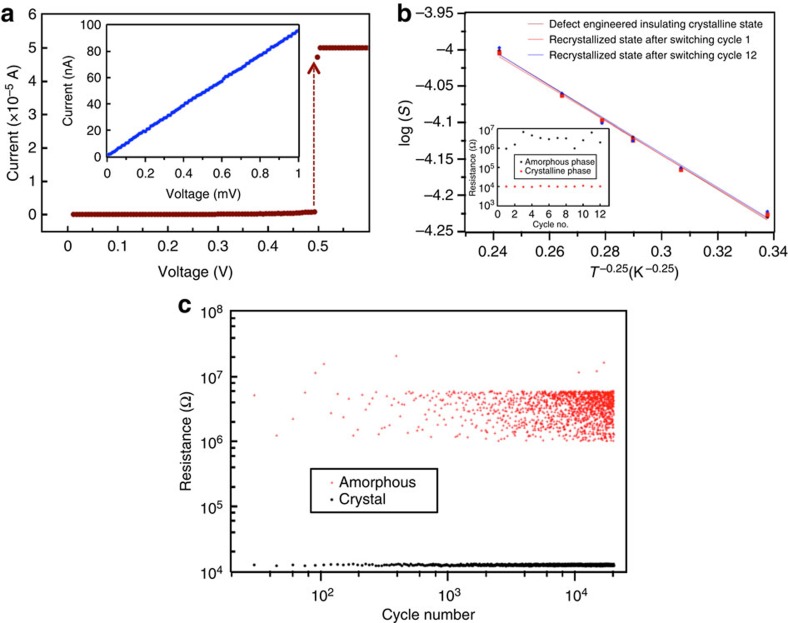
Recrystallization behaviour of defect-engineered GeTe nanowires switched to amorphous phase. (**a**) I–V sweep from 0 to 0.5 V on D1 (shown in Fig. 3) with compliance current (*I*_c_) set at 50 μA. Recrystallization followed by threshold switching occured at 0.5 V. Inset: low-bias resistance measurement on the recrystallized phase (∼10 kΩ). (**b**) Comparisons of the temperature dependence of conductivity measurements between as-engineered insulating crystalline phase, recrystallized phase after one cycle of switching and after 12 cycles of switching. All the recrystallized phases showed similar transport behaviour suggesting reliable and repeatable switching. Inset: data showing cycling between amorphous and crystalline phase. (**c**) Repeated cycling between RESET (26 μA, 100-ns pulse) and SET (1 V, 250-ns pulse) states for 20,000 cycles on D1.

To verify the reversible switching behaviour on the defect-engineered devices that displayed very low *j*_s_ (exposed at 3,600 μC cm^−2^ in Fig. 3), we examined the amorphous–crystal (SET) transformation via threshold switching^27^ by applying voltage controlled d.c. I–V sweeps. When the compliance current (*I*_c_) in the circuit was set to 50 μA, threshold switching followed by recrystallization through Joule heating of the amorphous phase occurred in all the devices at <1 V (see Fig. 4a for data on D1 and Supplementary Fig. 7 for data on D2). More importantly, as illustrated on D1 (Fig. 4b), the recrystallized phase after a few RESET/SET cycles showed similar resistance and temperature dependence of resistance, as the starting defect-engineered insulating crystalline phase. To demonstrate that the RESET state is stable, and does not fail via defect annihilation upon repeated switching, we cycled D1 between RESET (26 μA, 100 ns) and SET (1 V, 250 ns) for 20,000 cycles (Fig. 4c). We observed no appreciable changes in the resistance of the RESET state, validating the robustness of this low-power switching strategy. The band of resistance values in the amorphous phase, however, is a function of pulse width and amplitude. The combination of engineering carrier localization in crystalline phase of PCM, switching to the amorphous phase via the defect-based pathway, and recrystallizing the amorphous phase via Joule heating (pulsed mode or d.c.) is thus encouraging for ultralow-power phase-change memory operation. It must be noted that rigorous endurance tests demonstrating cyclability conforming to commercial standards (10^8^–10^9^ cycles) is a future endeavour.

### Demonstration of metastable intermediate states

**Figure 5:**
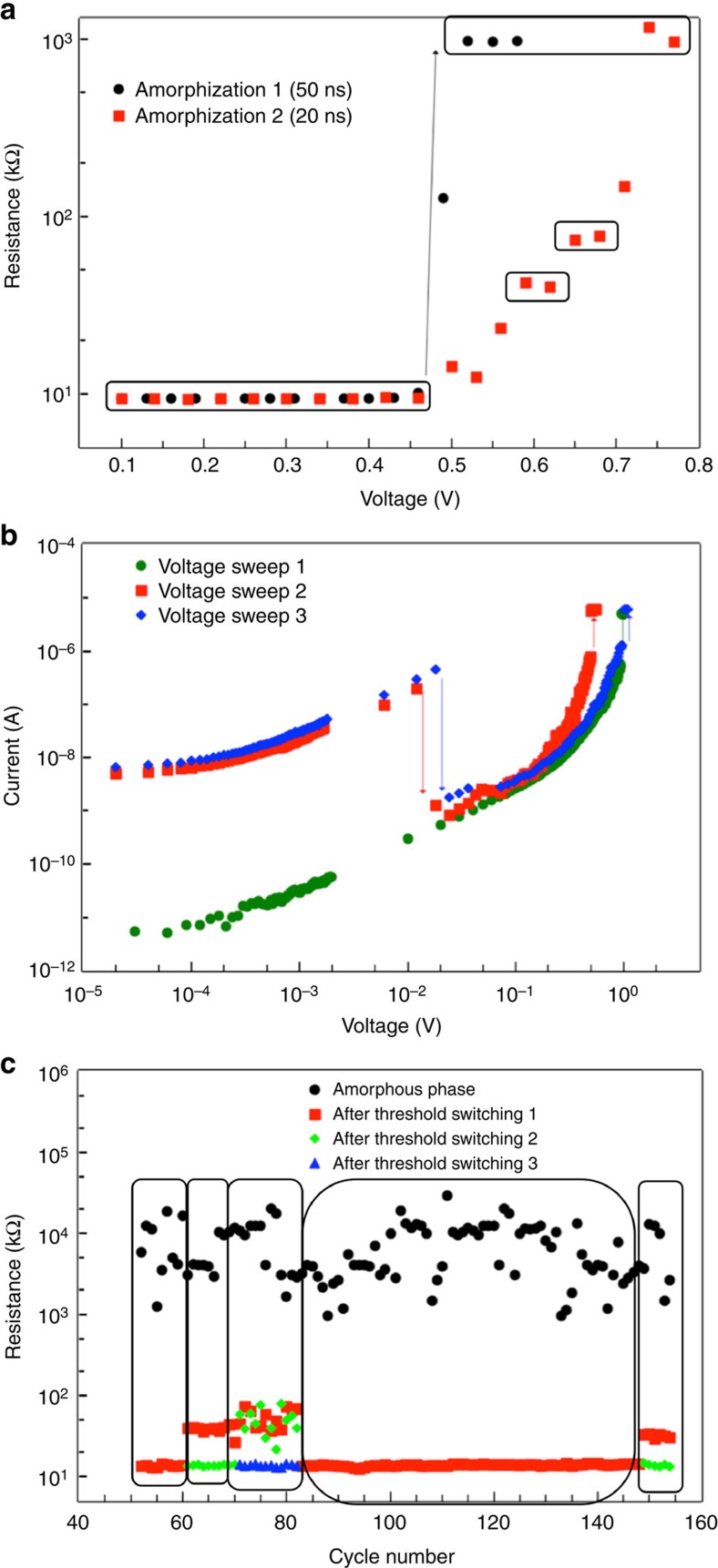
Accessing intermediate resistance states on GeTe nanowire devices defect engineered into insulating crystalline phase. (**a**) Programming curve on D1 for the RESET operation. When 50-ns pulses were applied, the transformation to the amorphous phase was sudden. With 20-ns pulses, the transformation happened gradually accessing several intermediate resistance states. Low-voltage, 20-ns pulses accumulates defects at a local region creating an intersecting defect template. Subsequent controlled addition of defects with higher amplitude pulses to this template increases the resistance of the device creating intermediate resistance states. States 1, 2 and 3, and amorphous phase (increasing order of resistance) are boxed. (**b**) Voltage sweep from 0 to 1 V (green) demonstrating a threshold-switching event of the amorphous phase to state 3 at <1 V with compliance current (*I*_c_) set at 5 μA. A second sweep starting from state 3 (red), revealing a drop in current at 0.01 V corresponding to amorphization event, and the amorphous phase subsequently transformed to state 2 after a threshold-switching event to state 2. Another voltage sweep from 0 to 1 V starting with state 2 (blue), again showing a drop in the current at 0.01 V, signifying amorphization (Supplementary Fig. 12a)—and the amorphous phase subsequently threshold switched and transformed to state 1. The arrows in the figure correspondingly indicate carrier-wind force assisted amorphization and threshold-switching events (**c**). Repeatable switching measurements, with every cycle consisting of a 100-ns, 26-μA pulse transforming state 1 to amorphous phase, followed by I–V sweeps until state 1 is eventually retrieved; and between every cycle *I*_c_ was randomly set to 50, 10 or 5 μA. For the first 60 cycles, *I*_c_ was set to 50 μA, for the next 10 cycles *I*_c_=10 μA. From 70 to 82 cycles, *I*_c_=5 μA, followed by 50 μA from 83 to 150 cycles. Further up to 160 cycles, *I*_c_=10 μA. When *I*_c_=50 μA, amorphous phase always switched to state 1 directly, and when *I*_c_=5 and 10 μA intermediate metastable states became accessible. Here the intermediate resistance states were created by controllably removing defects from the defect-templated region.

The defect-engineered crystalline states where carrier localization dominates transport, structurally corresponds to the entire nanowire device being replete with intersecting extended defects, or defect templates (Fig. 2a–d). Upon the application of electrical pulses to this state, more defects will migrate with momentum and energy transferred from the carriers, and accumulate at one (or more) of these templates increasing the local defect concentration^13^,^18^. A critical defect concentration locally would lead to the collapse of long-range order or ‘nucleation' of the amorphous phase. However, the question remains whether some intermediate metastable resistance states in crystalline phase can be accessed as the defect concentration at a template increases continuously towards a critical value at which amorphization occurs. To check whether defects can be controllably accumulated and whether the intermediate resistance states can be accessed, we reduced the pulse width of the voltage pulses used for programming defect-engineered insulating crystalline phase (resistance of 10 kΩ) to an amorphous phase, from 50 to 20 ns (Fig. 5a). Programming device D1 with 50-ns pulses abruptly nucleated the amorphous phase, suggesting that the energy transferred from 50-ns pulses is sufficient to migrate and accumulate defects beyond critical concentration at a region in the defect template. However, with 20-ns pulses, less energy is transferred to the defects, resulting in controlled defect accumulation and access to several intermediate metastable states in the crystalline phase (see Supplementary Fig. 8 for TEM data) whose resistance increases with increasing defect concentration at the template (thermal stability of intermediate and amorphous phases is discussed in Supplementary Note 9 and Supplementary Fig. 9). Similar intermediate states were observed in all the other devices exposed to a dosage of 3,600 μC cm^−2^ upon programming them with 20-ns pulses (Supplementary Fig. 10). For the discussion that follows we will refer to the representative electronic states in D1 in the crystalline phase with resistances of ∼10, 40 and 70 kΩ as states 1, 2 and 3, respectively (Fig. 5a).

To understand whether these intermediate resistance states can be reversibly obtained starting from the amorphous phase, we recrystallized the amorphized device D1, via d.c. I–V sweeps, setting a very low compliance current (*I*_c_) of 5 μA. As shown in Fig. 5b, upon a voltage sweep from 0 to 1 V (green data), the amorphous phase first transformed to an intermediate resistance state (70 kΩ, state 3). Upon a second voltage sweep from 0 to 1 V on state 3, we observed a sudden drop in current at 0.02 V (0.1 μA), followed by a threshold-switching event to another intermediate state (red data in Fig. 5b) with resistance of 40 kΩ, state 2. Another voltage sweep from 0 to 1 V on state 2 showed a similar drop in current at 0.02 V, followed by a switching event to the starting electronic state, state 1 (10 kΩ, blue data in Fig. 5b). To ensure reliability in the formation of all the demonstrated states, we switched these devices for 160 cycles, where every cycle involved the following steps: switching state 1 to a high-resistance amorphous phase by the application of a 100 ns, 26 μA pulse, and switching back to state 1 from the amorphous phase via sweeping d.c. voltage from 0 to 1 V, multiple times, if necessary (depending on the value of *I*_c_). We changed the *I*_c_ between cycles to confirm the dependence of formation of intermediate states on *I*_c_ (Fig. 5c). When *I*_c_ was set to 50 μA, we observed only two states: a high-resistance amorphous state (>1 MΩ), and state 1, a low-resistance crystalline state (∼10 kΩ). However, when *I*_c_ was 10 μA, we consistently observed amorphous state first transforming into an intermediate resistance state (∼40 kΩ) with the first voltage sweep, and then to state 1 with another voltage sweep from 0 to 1 V. Upon further reducing *I*_c_ to 5 μA, we observed amorphous phase to state 1 transformation in every cycle requiring three voltage sweeps from 0 to 1 V, with the first two voltage sweeps accessing two intermediate resistance states (between 35 and 80 kΩ with some variability), and the final sweep transforming these intermediate states to state 1. It must be noted that the variability of resistance of the intermediate states (Fig. 5c) upon cycling (with *I*_c_=5 μA), may not currently conform to commercial standards for multistate memory applications, and need to be addressed and improved in further studies. Nevertheless, these findings provide a proof of concept that intermediate states can reliably obtained starting from both crystalline phase and an amorphous phase, with the controlling parameters being the pulse width and *I*_c_ (Joule heating following threshold switching), respectively.

From these results, the mechanism for amorphous–crystal transformation in the defect-based pathway becomes clear. Following amorphization, most of the nanowire has a background density of pre-induced defects, and a local region has a higher defect concentration (defect template). The amorphous region is a part of this template where the defect concentration exceeds a critical value, and it cuts across the cross-section of the nanowire^13^,^18^ (Supplementary Fig. 11a–c for TEM images). Transformation from the amorphous phase to any crystalline state involves threshold switching followed by recrystallization of the amorphous region, and subsequently a reduction of defect concentration in the rest of the template through homogenization of defects (to the background concentration) via Joule heating. Thus following recrystallization, the degree of defect homogenization can be controlled via *I*_c_, and this provides access to several intermediate resistance states in the crystalline phase (Supplementary Note 10).

### Amorphization of intermediate resistance states via d.c. current

Another subtle feature in d.c. I–V switching behaviour of intermediate states (states 2 and 3 of D1) is the sudden drop in current (increase in resistance) at very low currents (0.1 μA in Fig. 5b, red and blue curves). This event corresponds to intermediate states first transforming to an amorphous phase, a permanent structural change (Supplementary Fig. 12a,b). It is consistent with the understanding that the momentum (and energy) transfer from carriers to defects at 0.1 μA is sufficient to migrate more defects to the already existing defect-template region in the intermediate states, thus increasing the defect concentration beyond a critical limit to nucleate the amorphous phase. These results demonstrate that crystal–amorphous transformation in the defect-templated pathway is a solid-state transformation, assisted by carrier-wind force and strong carrier–lattice coupling (Supplementary Note 11).

### Transport properties of various observed resistance states

**Figure 6:**
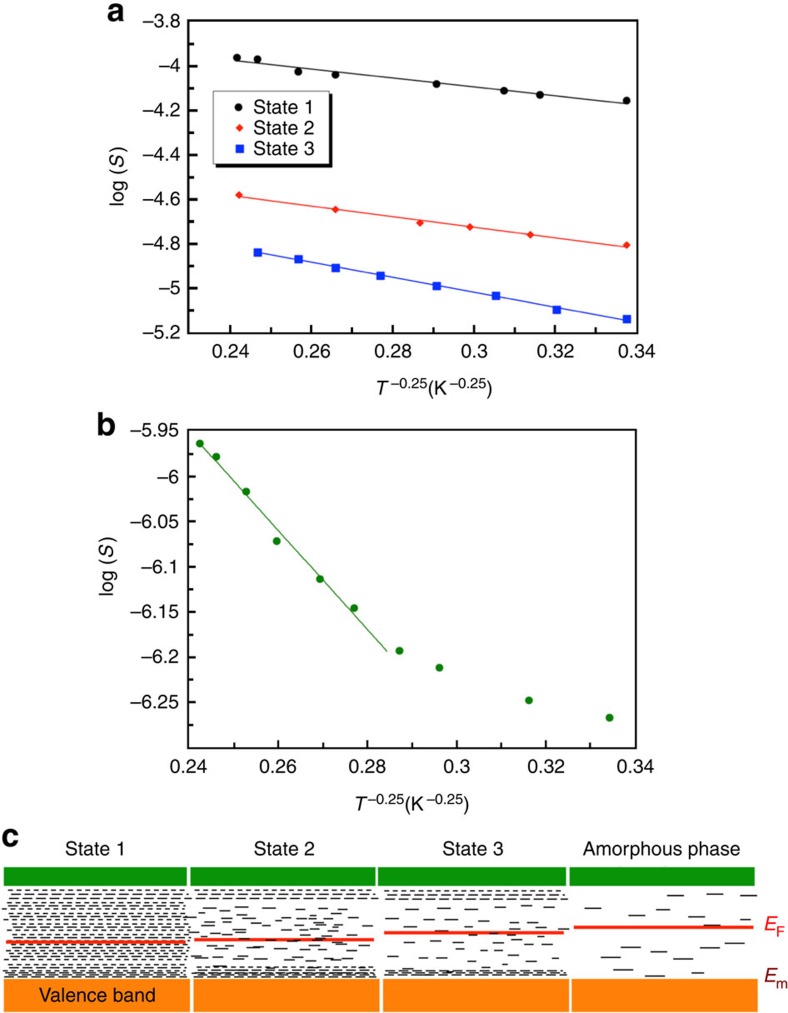
Transport measurements on various observed resistance states in defect-engineered GeTe nanowires. (**a**) *T*^−0.25^ versus log(*S*) plots, where *S* is the conductance—showing that states 1, 2 and 3 exhibit VRH conduction behaviour with slopes (*A*) becoming steeper from 1 to 3. *A* in states 1, 2 and 3 are −2, −2.4 and −3.4 K^0.25^, respectively. (**b**) Conduction behaviour of the amorphous phase plotted as log *S* versus *T*^−0.25^, showing VRH behaviour at high temperatures (*A*=−5.1 K^0.25^), and a deviation from VRH at low temperatures most likely due to the formation of paired carrier traps and *E*_F_ pinning. (**c**) Schematic band diagrams showing the relative position of Fermi level (*E*_F_) and density of single-electron trap states at *E*_F_ (*N*(*E*_F_)) in all the observed states. From state 1 to the amorphous phase, Fermi level progressively moves into deeper traps until *E*_F_ gets pinned to the mid-gap in the amorphous phase, and the single-electron trap density (*N*(*E*_F_)) decreases at the cost of increasing charged (paired) traps.

To understand the differences in various observed resistance states (states 1–3 and amorphous phase) from an electronic viewpoint, we performed temperature-dependent resistance measurements on D1 prepared in these states (Fig. 6a,b). States 1, 2 and 3 (∼10, 40 and 70 kΩ, respectively) showed VRH conduction, where conductance *S* depends on temperature as *S*=*S*_0_ exp(−*AT*^−0.25^), with *A*, the temperature-independent prefactor, increasing from 2 to 2.4 to 3.4 K^0.25^ from states 1 to 3 (Fig. 6a). *A*=[*α*^3^/*kN*(*E*_F_)]^0.25^, where *α* is the inverse of carrier localization length, *k* is the Boltzmann constant and *N*(*E*_F_) is the density of single-electron trap states at the *E*_F_ (ref. 28). The increase in value of *A* from states 1 to 3 could be a result of decrease in localization length (or increase in *α*) or a decrease in *N*(*E*_F_) or both. On the other hand, the conduction characteristics of the amorphous phase (Fig. 6b) showed VRH behaviour at high temperatures (>150 K), with *A*=5.1 K^0.25^; and deviated from VRH behaviour (log(*S*) proportional to *T*^−0.25^) at low temperatures (Supplementary Fig. 13). This behaviour is different from that of typical melt-quench amorphous phase, which shows activated conduction via emission of carriers into delocalized states at high temperatures and VRH at low temperatures^29^ (Supplementary Note 12). It will be an interesting future direction to rigorously study the nature of the observed amorphous phase and understand device-related issues such as resistance drift in comparison with the conventional melt-quench amorphous phase.

The schematics of band structure shown in Fig. 6c explain the observed conduction characteristics (Fig. 6a,b) of various representative states. In state 1, *E*_F_ is above the mobility edge (*E*_m_) surrounded by a large density of single-electron traps *N*(*E*_F_). Progression from state 1 to state 3 by adding more defects either shifts the *E*_F_ towards the mid-gap, which reduces *N*(*E*_F_) and localization length, or creates more paired trap centres, which reduces *N*(*E*_F_). Finally, in the amorphous phase, *E*_F_ is pinned to the mid-gap^30^,^31^, with *N*(*E*_F_) at its minimum value. In the reverse process (SET), by controlled homogenization (removal) of defects from the defect-template region via Joule heating, *E*_F_ moves back towards the mobility edge, and *N*(*E*_F_) starts to increase, accessing all the crystalline intermediate resistance states. Pulse width (Fig. 5a) and compliance current (Fig. 5c) can be used to control the addition and homogenization of defects respectively. It is important to note here that multiple resistance states in PCM reported in earlier works^30^,^31^,^32^,^33^,^34^ were created by controlling the relative volumes of the amorphous and crystalline states, the only two physically different states, and are fundamentally different from the multiple resistance states in this work.

## Discussion

We demonstrated that defect-engineered crystalline GeTe with dominant carrier localization effects transforms to an amorphous phase at current and power densities significantly lower than some of the best reported low-power devices operated through melt-quench strategy^6^. These results emphasize the importance of carrier–lattice coupling in the insulating state in carrying out energy efficient amorphization. Our devices displayed good reversibility and endurance, suggesting new strategies for potentially mitigating the issue of chemical segregation and heat-based device degradation problems, common in melt-quench approach^11^. The recent discoveries of localization effects^35^ and defect-templated amorphization^18^ in Ge–Sb–Te alloys suggests that defect-engineering approach could be applied to other well-known PCMs too. In addition, with our demonstration of scaling of switching properties in the defect-based approach and multistate switching, we believe that nanoscale PCM structures (mushroom, sidewall, pillar or confined architectures)^5^,^36^ engineered into electronic states in crystalline phase that show localization behaviour in transport will be promising for ultralow-power memories and novel computation strategies^29^,^30^.

## Methods

### Synthesis of GeTe nanowires

GeTe nanowires were synthesized using metal catalyst-mediated vapour–liquid–solid process, where bulk GeTe powder (99.9%, Alfa Aesar, melting temperature, 724 °C) was placed at the centre of a tube furnace. Silicon oxide substrate evaporated with Au film (8 nm) and subsequently annealed at 720 °C for 10 min was placed on the downstream side of the furnace (∼15 cm away from the middle). The furnace was heated to 400 °C at a carrier gas (Ar) flow rate of 100 s.c.c.m. and a pressure of 10 torr, and maintained so for 10 h before the furnace was slowly cooled to room temperature.

### Device fabrication

Multiple electrode devices were fabricated using electron-beam lithography. Nanowires were dry transferred onto an insulating substrate with pre-patterned markers. Three layers of PMMA 495 A4 and three layers of PMMA 950 A2 were spin coated and baked (180 °C) following which electron-beam lithography, metallization (Ti/Au: 50/100 nm) and lift-off procedure were performed. The devices were subsequently annealed at 350 °C, and a 30-nm SiOx film was conformally deposited by plasma-enhanced chemical vapour deposition.

### Ion irradiation

The devices thus fabricated were irradiated with 2 MeV He^+^ ions, (beam area: 5 × 5 mm) in a tandem accelerator (NEC minitandem ion accelerator). Substrates were aligned perpendicular to the beam, and ion bombardment was performed until a cumulative prescribed dosage was reached, with ion current maintained below 30 nA (as measured from picoammeter). Dosage was calculated as *It*/*A*, where *I* is the instantaneous ion current, *t* is the time of exposure and *A* is the area of the beam area (5 × 5 mm). We verified using SRIM (Stopping and Range of Ions in Matter) software that He^+^ ion exposure and knock-on damage is uniform throughout the thickness of the nanowire devices (Supplementary Note 1).

### Electrical testing

Temperature–resistance measurements were performed in the Lakeshore TTPX cryogenic probe station, and resistance measurements were carried out via I–V sweeps at very low bias (−2 to 2 mV) using Keithley 2,602 (I–V analyser/source meter). Switching and endurance tests were performed using Keitheley 3,401 for pulse generation, 2,602 (I–V analyser) for resistance measurement after the application of the pulse and Keithley 2,700 as the data acquisition system. The shapes of the voltage pulses generated and dynamic current response produced were verified using a 500 MHz Tektronix DPO3052 digital oscilloscope. Applied voltage pulse was measured by connecting the device in parallel to the 50 Ω input channel 1 of the oscilloscope. The current response was measured by measuring the voltage drop across a 50-Ω resistor connected in series with the device, and in parallel with a second 50 Ω input channel of the oscilloscope.

## Additional information

**How to cite this article:** Nukala, P. *et al*. Ultra low-power switching via defect engineering in germanium telluride phase-change memory devices. *Nat. Commun.* 7:10482 doi: 10.1038/ncomms10482 (2016).

## Supplementary Material

Supplementary Information Supplementary Figures 1-13, Supplementary Table 1, Supplementary Notes 1-12 and Supplementary References
