# Supplementary material for: Ultralow-power switching via defect engineering in germanium telluride phase-change memory devices
Source: Nat Commun. 2016 Jan 25;7:10482. doi: 10.1038/ncomms10482 (PMC4737760; doi:10.1038/ncomms10482)
Supplement: Supplementary Information — Supplementary Figures 1-13, Supplementary Table 1, Supplementary Notes 1-12 and Supplementary References [file ncomms10482-s1.pdf]

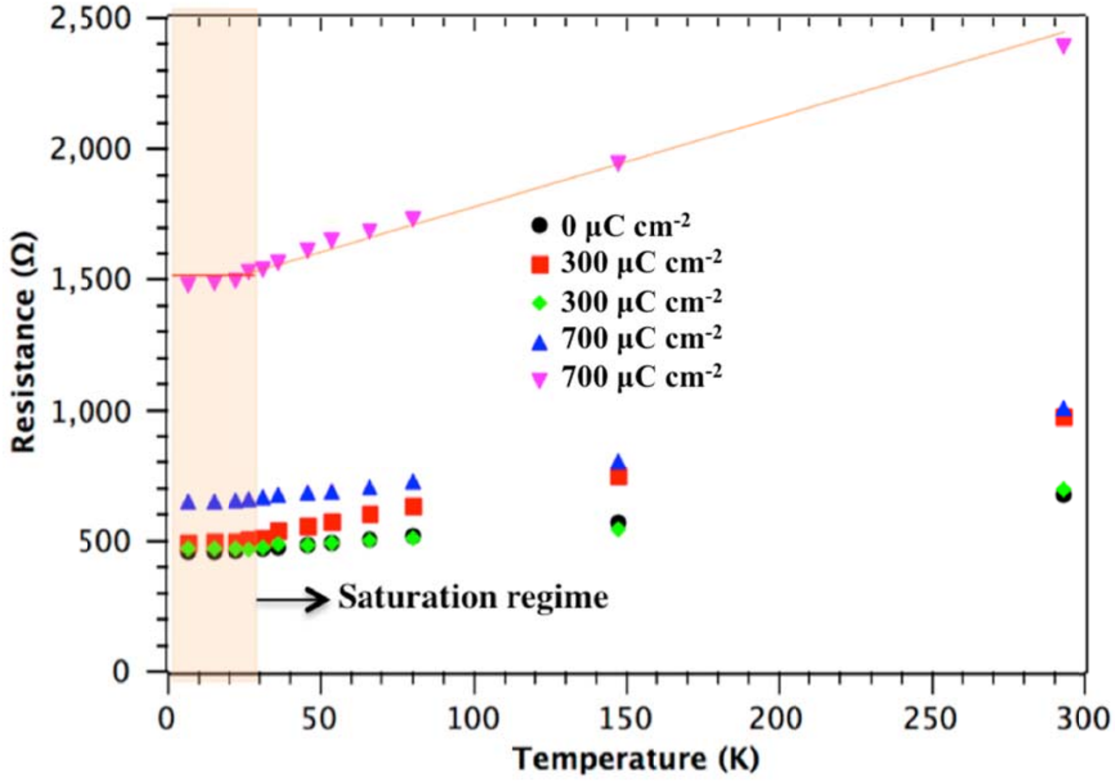

Supplementary Figure 1: Raw temperature-resistance plots of a few different nanowire devices in the metallic phase exposed to different dosages of He^{+} ions. Note the saturation of resistance below 30 K, and its linear dependence on temperature above 30 K in all these representative devices.

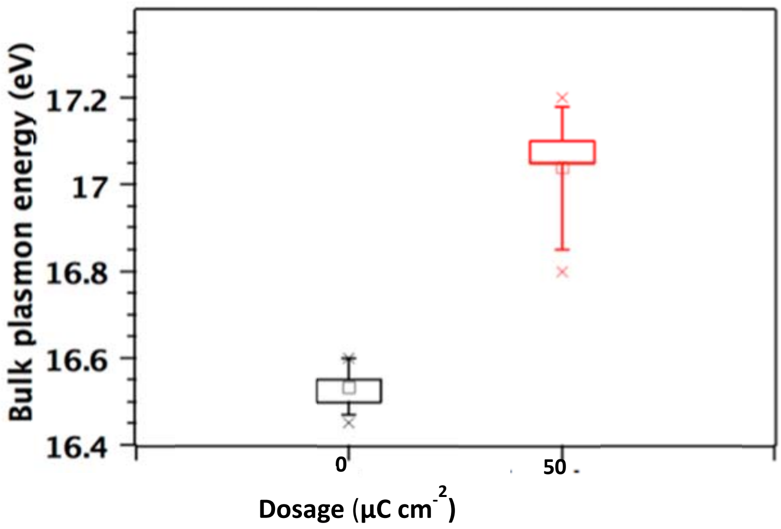

Supplementary Figure 2: Box-plots of STEM EELS data of plasmonic peak energy on 11 different nanowire devices before irradiation and after irradiation with 50 µC cm^{-2} of 2 MeV He^{+} ions. Plasmon peak energy at no dosage is 16.5 eV with a standard deviation of 0.1 eV, whereas at 50 µC cm^{-2}, it is 17.1 eV, with a standard deviation of 0.3 eV.

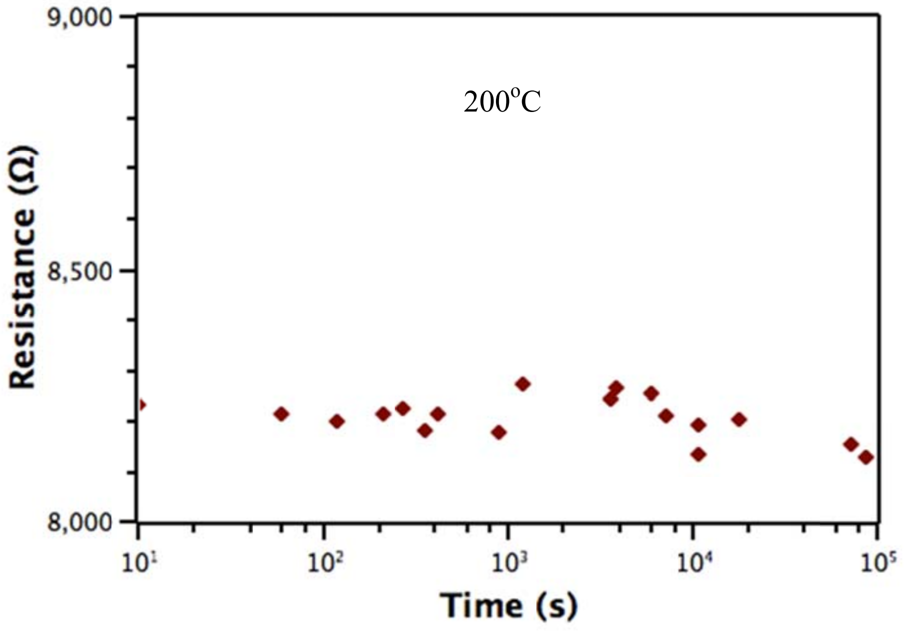

Supplementary Figure 3: Plot showing that the resistance of as-engineered insulating crystalline phase does not change with time at 200 ^{o}C for at least 36 hours, demonstrating its thermal stability.

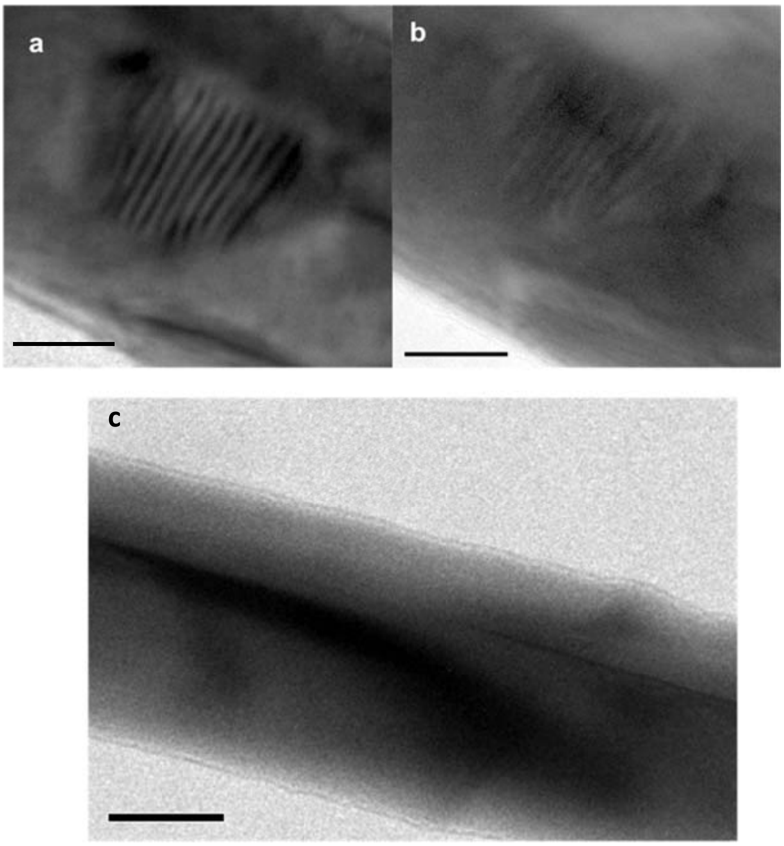

Supplementary Figure 4: (a) Bright-field TEM image of one of our nanowires irradiated with 2.2 x10^{16} ions cm^{-2} fluence of He^{+} ions. No He bubbles can be observed. (b) The same region of the nanowire was imaged in BF mode by 1000 nm of under-focusing. No voids can be observed. (c) BF image on another nanowire showing no bubbles. Scale bar in all the images is 50 nm.

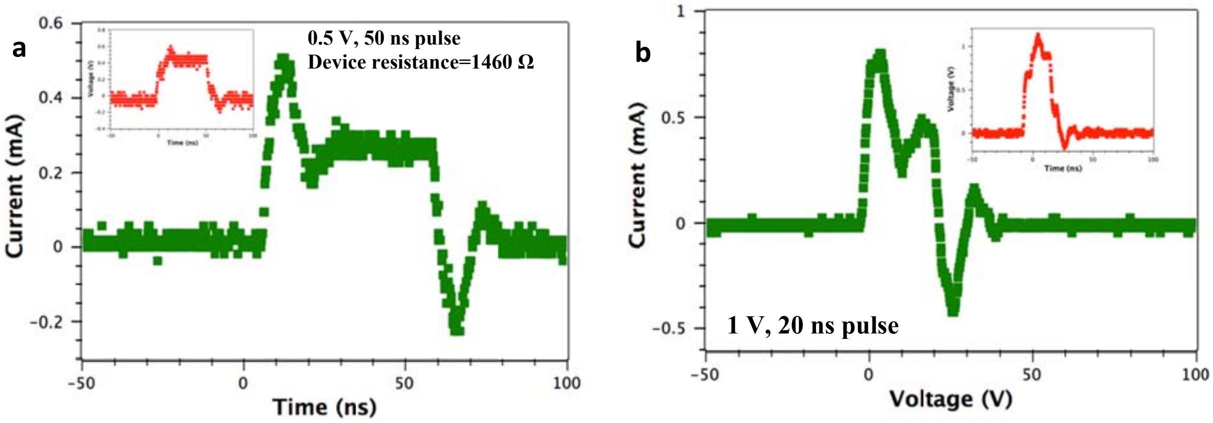

Supplementary Figure 5: Current response (green) for an applied voltage pulse (red) of 0.5V, 50 ns in (a) and 1V, 20 ns pulse in (b).

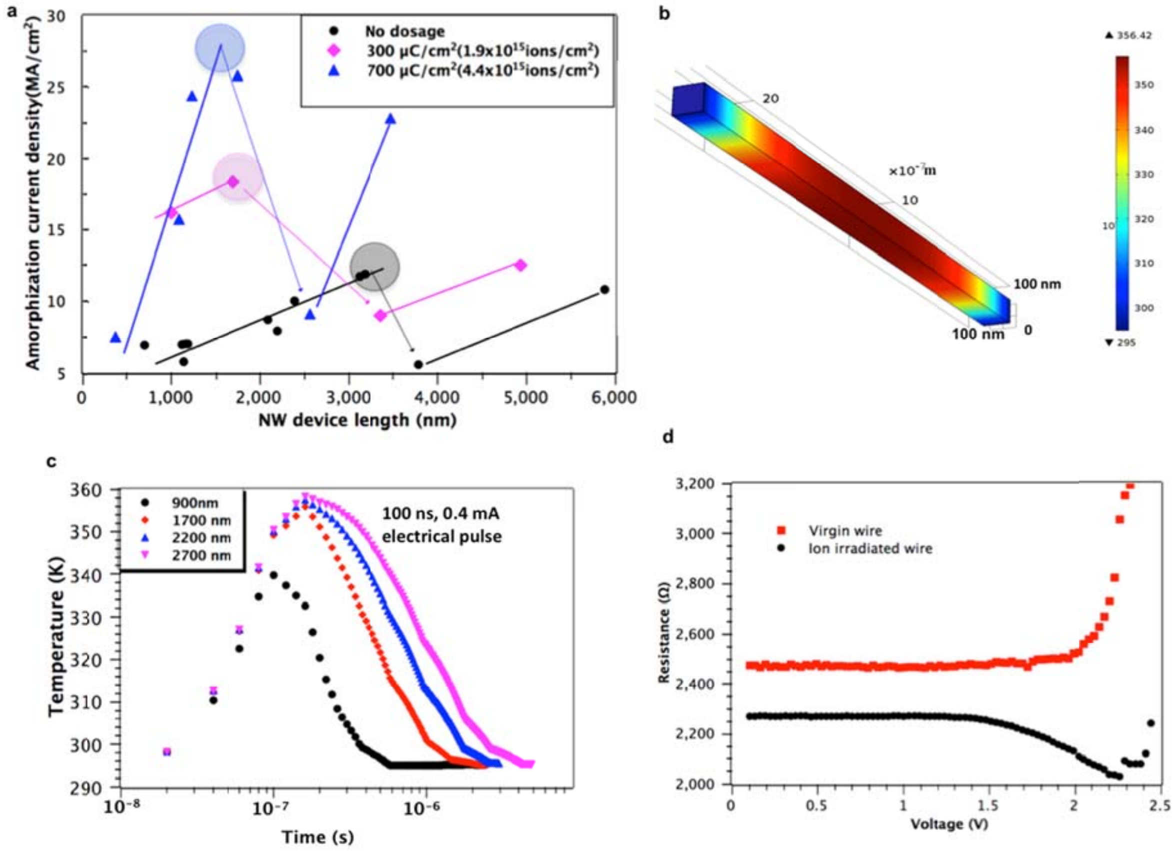

Supplementary Figure 6: (a) Switching current density vs. length of the device plots at low dosages, 0 (black), 300 (magenta) and 700 (blue) µC cm^{-2}. After a critical length l_c (shaded), the switching current suddenly drops at all these dosages. (b) Spatial temperature profile upon the application of 0.4 mA, 100 ns current pulse in a nanowire, which is 2.2 µm long, and 100 nm in diameter, 20 ns after the pulse is withdrawn. (c) Temperature vs. time profile of heat shock at the central point of the nanowires with differing lengths and 100 nm diameter; when a 100 ns, 0.4 mA current pulse is applied- calculated using finite element modeling; showing the severity of heat shocks in shorter devices than longer ones. (d) Comparison between programming curves in non-irradiated device and devices irradiated at dosages up to 700 µC cm^{-2}, suggesting that pre-induced defect annealing/reorganization in irradiated devices (at low dosages) requires extra work done on the system, and this manifests itself as higher switching currents.

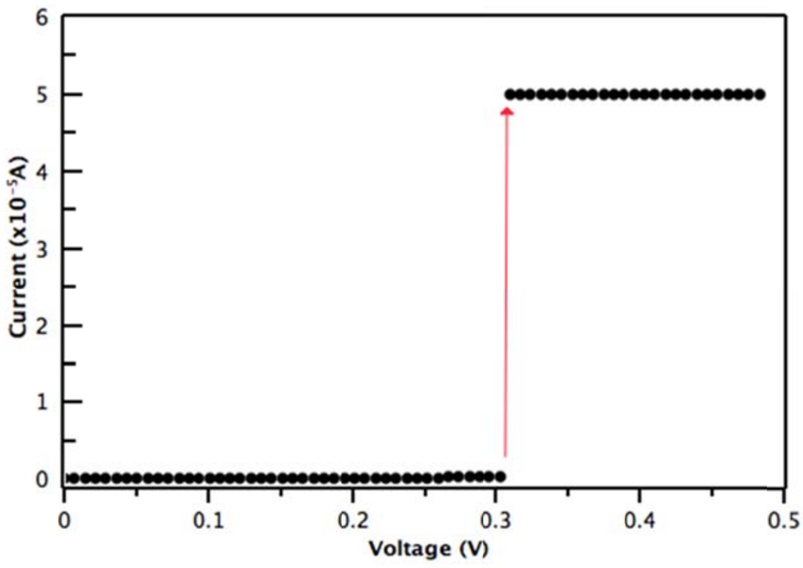

Supplementary Figure 7: Threshold switching on device (320 nm x 80 nm x 80 nm) which amorphized at a low current (density) of 8 μA (0.13 MA cm^{-2}).

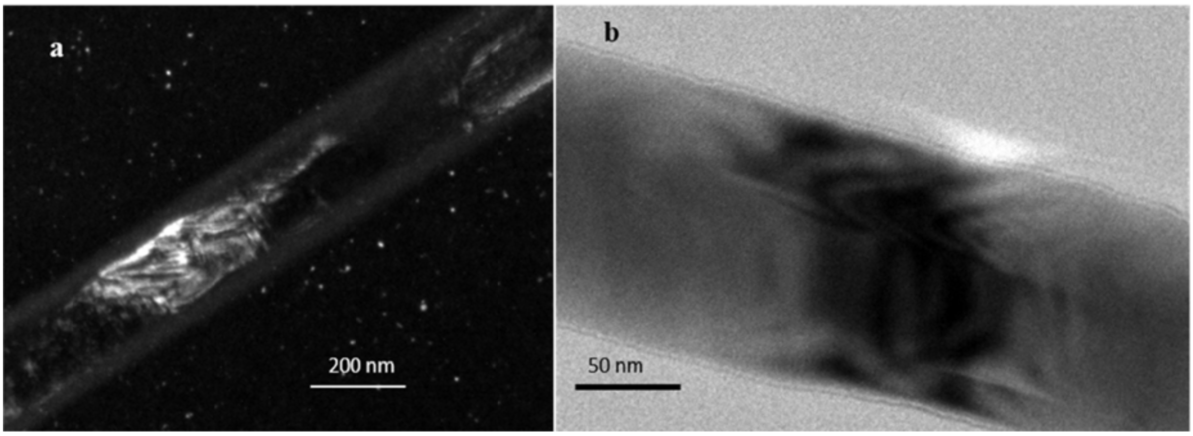

Supplementary Figure 8: (a) Dark-field TEM image of a defect-engineered device stabilized in an intermediate state. We can clearly observe the contrast of large concentration of defects (exceeding the background) in a local region. (b) Bright-field TEM image of an intermediate state in another defect-engineered device showing similar microstructure as the device in (a).

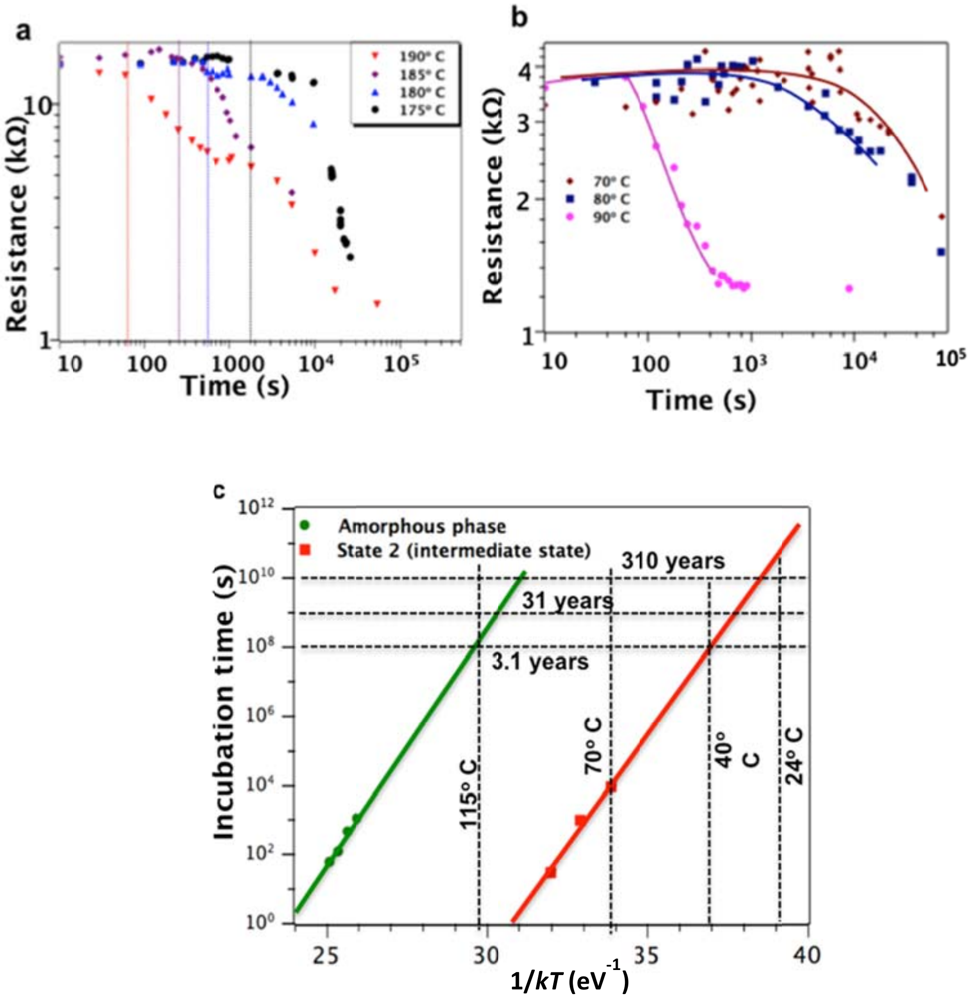

Supplementary Figure 9: (a) Resistance–time plots (retention measurements) on amorphous phase, isothermally held at 190, 185, 180, 175 ^{o}C respectively. The incubation times (retention times) at every temperature are indicated as dotted lines. (b) Retention measurements on an intermediate state (state 3) showing its stability at 70, 80 and 90 ^{o}C respectively. (c) Arrhenius plots of incubation times of both amorphous phase and intermediate phase extracted from (a) and (b) respectively. Extrapolations to desired operations temperature give the thermal life times of these states at that temperature. Vertical dotted lines are constant temperature lines, and horizontal dotted lines are constant time lines. These extrapolations show that the intermediate state (2) is stable for 3.1 years at 40 ^{o}C, and that the amorphous phase has a large thermal stability.

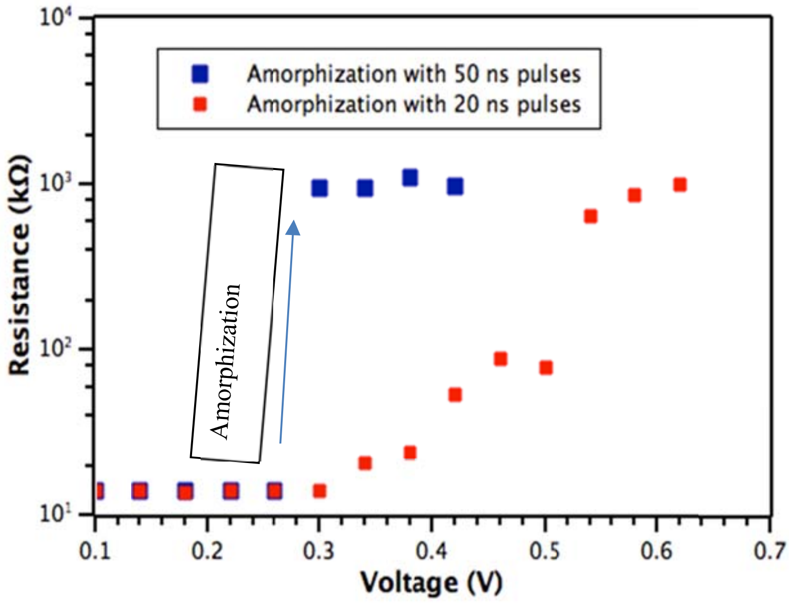

Supplementary Figure 10: Programming curve on another device showing the formation of intermediate states with the application of 20 ns pulses, and abrupt switching at 23 µA upon applying 50 ns pulses on a device defect-engineered at 3600 µC cm^{-2}.

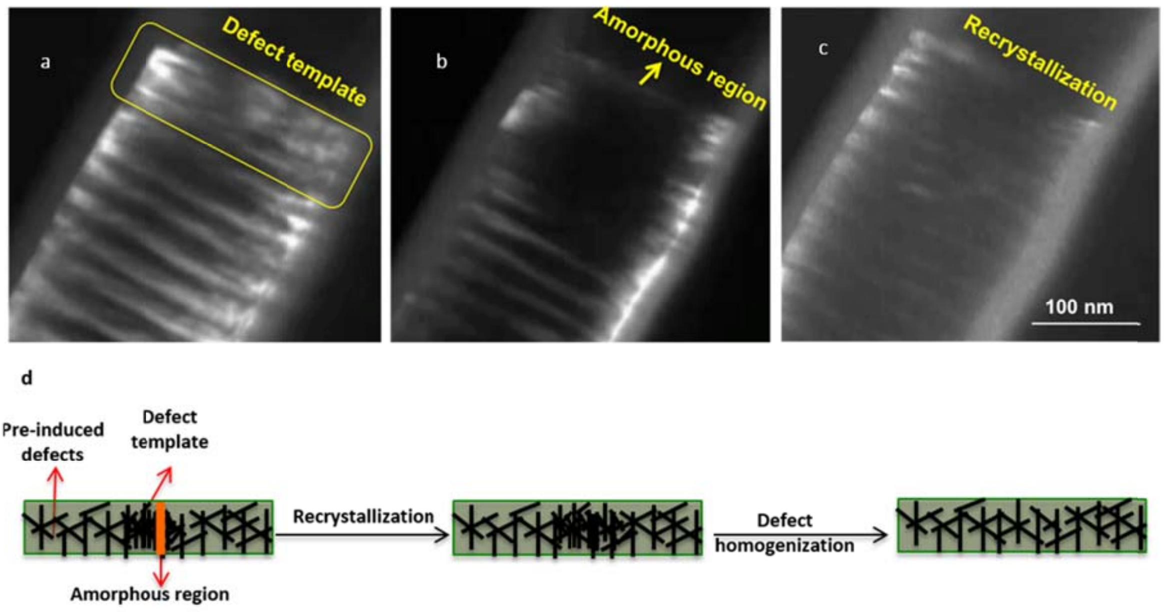

Supplementary Figure 11: (a) Dark-field (DF) TEM image just prior to amorphization revealing the presence defect-template, which leads to amorphization with more addition of defects to this template. (b) DF-TEM of the same device shown in (a), post-amorphization. The amorphous mark contrast can be clearly identified (dominating over the defect-template that surrounds it). (c) Recrystallization event, showing similar contrast all across the device, suggesting homogenization of defects from the template to a background concentration. (d) Schematic showing the mechanism of switching amorphous phase to starting crystalline state.

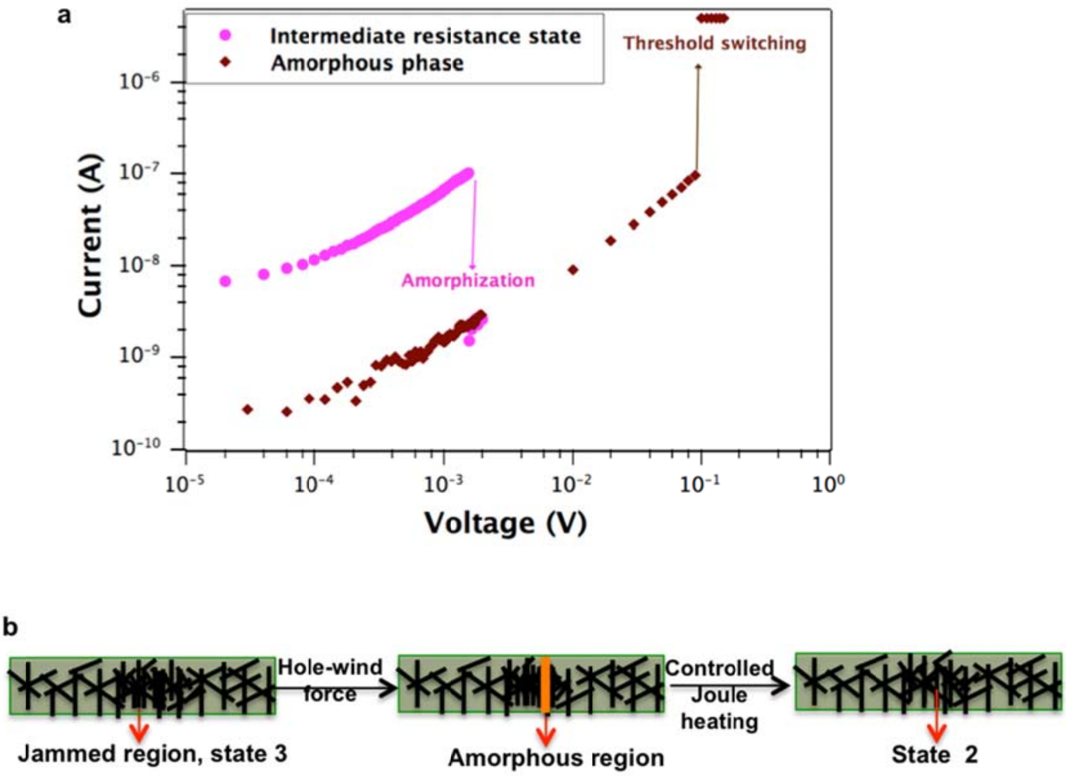

Supplementary Figure 12: (a) (Magenta) Voltage sweep on an intermediate state until 0.002 V showing a drastic drop in the current at approximately 0.001 V (0.1 μA). (Brown) A second voltage sweep confirming that the drop in current in the earlier sweep is because of an amorphization event, and is not a field dependent reversible effect; and in this voltage sweep, the amorphous phase is threshold switched to another intermediate state (I_c=5 μA). (b) Schematic illustrating the changes in defect configurations during the various switching processes. Nanowire in the intermediate state has a background defect density (corresponding to that of state 1) in most of the nanowire, and a higher density in a very local region. Hole-wind force at a d.c. current of 0.1 μA collects defects in this local region, such that their concentration exceeds the critical limit to amorphize the region. Upon Joule heating the amorphous phase in a controlled fashion (controlling compliance current), another intermediate state can be created.

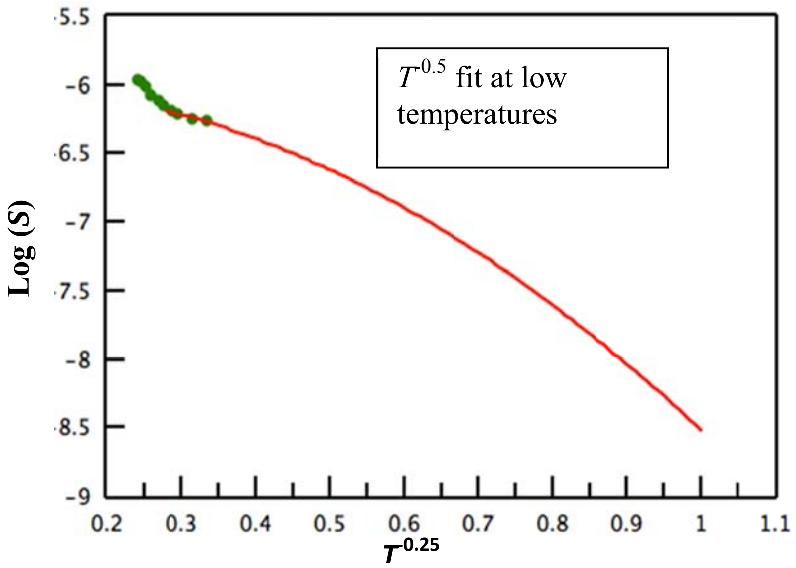

Supplementary Figure 13: Fit showing a possible E-S conduction at low-temperatures in our amorphous phase. The same few data points can also be fit to $T^{-0.25}$, and because of this ambiguity we did not show these fits in the manuscript.

	RESET current [density] (μA [MA cm^{-2}])	Active volume (nm^3)	Power density for switching (mW μm^{-3})
PCM with CNT electrodes (melt-quench) [Xiong et al., Science, 332, 568 (2011)]	5 [50]	35 x 3 x 3	~80,000
Phase-change bridge (melt-quench) [Y.C.Chen, IEDM, 2006]	100 [167]	80 x 20 x 3	~80,000
This work (defect-templated)	8 [0.13]<br>14 [0.29]<br>23 [0.62]<br>26 [0.26]	320 x 80 x 80<br>900 x 70 x 70<br>2600 x 60 x 60<br>750 x 100 x 100	~2.1<br>~0.83<br>~0.6<br>~0.65

Supplementary Table 1: RESET power and current density comparisons between melt-quench strategy of amorphization and the current defect-engineered, defect-templated methodology. RESET power densities (evaluated as i^2R/Volume) for devices prepared in states with carrier localization effects in this work are at least 5 orders of magnitude less than those reported for devices switched via melt-quench pathway.

Supplementary Note 1: Stopping and Range of Ions in Matter (SRIM) calculations in GeTe for He^+ ions :

At high incident energy of He^+ ions, the particle energy decreases along the penetration through the alloy, and this is parametrized by stopping power (defined as $S=dE/dx$: energy lost per unit penetration depth). At low penetration depths, the stopping power is very uniform, suggesting that the very little fraction of this energy loss occurring due to nuclear interactions (knock-on damage) is also very uniform. Using SRIM, we verified that the stopping power of GeTe alloy for 2 MeV He^+ particles is very uniform in the thickness range of nanowire devices used in this work (from 60 to 200 nm), with only 2% of the incident energy lost in a 200 nm thick nanowire (2 MeV to 1.96 MeV). This very little energy loss also means that almost all the incident He^+ ions penetrate through the sample without getting implanted in the form of He bubbles. Furthermore, the knock-on damage due to ion-bombardment is homogeneous through the nanowire, without any influence from the abrupt energy loss.

Supplementary Note 2: Extrapolation of saturation resistivity (ρ_0) and temperature coefficient of resistivity (TCR) from resistance-temperature plots in the metallic phase

Supplementary Figure 1 shows temperature dependence of resistance of some representative devices in the metallic phase radiated by He^+ ions at different dosages (0, 300, 700 μC cm^{-2}). In all these devices in the metallic phase resistance increases with temperature beyond 30K where phonon-carrier scattering dominates transport, and saturates below 30K where carrier-defect scattering is predominant. So, the resistance extrapolated to 30 K is the saturation resistance (R_s) from which saturation resistivity (ρ_o, see Fig 1a) is calculated as $\rho_o = R_s A/l$, where A is the cross-sectional area of the nanowire, l is its length. The slope of the resistance-temperature plots when resistance increases linearly with temperature is the temperature coefficient of resistance, from which TCR is calculated by multiplying the slope with a geometric factor A/l. Note that the nanowire resistance is calculated by subtracting the contact resistance extracted from a multi-probe measurement from the device resistance.

Supplementary Note 3: Plasmonic spectroscopy, carrier counting and explanation of TCR trends with dosage in the metallic phase:

As per ref. 1, an approximate expression for TCR in metallic phase considering the weak-localization corrections from defects, is given as $TCR = \frac{2\pi m}{ne^2}\frac{N_0 I_0^2}{M\omega_D^2}\left[1-\frac{3}{(k_f l)^2}\right]$, where $k_f l$ (k_f- Fermi vector and l- mean free path of electrons) decreases with increasing defect density resulting in the decrease of TCR. However, while e –electron charge, and I_o–matrix element for electronic plane wave states, are material independent, and M– ionic mass, is not influenced by defects, N_0, ω_D – density of states at Fermi level and the Debye frequency, respectively, vary in a complicated manner with the defect-density; the increase in m – effective carrier mass, and the decrease in n – the carrier concentration, with increasing defect density can result in the increase in TCR, a behavior reflected at very low dosages (up to 50 μC cm^{-2}, Fig. 1b).

Increase in m with dosage is trivial to understand, however, to verify if carrier concentration (n) decreases upon ion irradiation we performed plasmonic spectroscopy using energy electron loss spectroscopy (EELS) in scanning transmission electron microscopy (STEM). The energy of the bulk plasmon resonance is directly dependent on the bound electron density in a material as $\omega_p{}^{0.5} \sim N_{bound-electrons}{}^{2}$, and an increase in the energy of plasmonic resonance corresponds to an increase in the bound electron density or a decrease in hole-carrier concentration. In Supplementary Figure 2, we show that upon irradiation at low dosages (50 μC cm^{-2}) on 11 different nanowires, the bulk plasmon peak on an average increases from 16.5 eV to 17.1 eV, indicating a decrease in the hole-carrier concentration upon irradiation by 2% (i.e. $\left(\sqrt{\frac{17.1}{16.5}}-1\right)\times 100$). In GeTe, since Ge vacancies are responsible for large hole concentration (10^{19}-10^{21} per cm^3)[3], decrease in hole concentration structurally corresponds to a decrease in Ge vacancy concentration, which is to be expected in ion-irradiation where vacancies supersaturate to form extended defects (Fig. 2 in the manuscript).

Supplementary Note 4: Stability of the insulating crystalline state (state 1) engineered by pre-inducing defects

We measured the resistance of the as-engineered insulating crystalline state at 200 °C as a function of time to study the thermal stability of this phase. As shown in Supplementary Figure 3, there was no change in the resistance of this phase atleast for 36 hours at 200 °C; and this suggests that thermal degradation of this phase via pre-induced defect annealing during memory operation is a non-issue. The SET and RESET cycling data for ~20000 times on a defect-engineered device shown in Fig. 4c of the manuscript, is a further testimony to the stability of defects in the defect-engineered insulating crystalline phase. The SiO_2 conformal coating, we presume plays an important role interms of removing any surface sinks for the extended defects, giving rise to this extraordinary stability.

In contrast, the insulating phase created through electrical pulses in GeTe demonstrated in our earlier work (ref. 4) is not stable beyond 70°C. The difference arises from the fact that in this work, defects are pre-induced homogeneously in all parts of the nanowire; whereas in the insulating state of ref. 4, electrical pulses accumulate the defects at a local region, with relatively clean metallic crystals surrounding it[13] (as is the case with intermediate states in this work discussed in Supplementary Note 6). Upon heating to 70°C (ref. 4), these non-homogenously distributed defects tend to homogenize to a background defect-density, explaining the low thermal stability of insulating phase in ref. 4 (created by electrical pulses from a defect-free devices).

Supplementary Note 5: He bubbles and associated voids during He^+ ion irradiation

It is well known from literature that the implanted He^+ ions cannot stabilize as such in any material but rather transform to He bubbles and escape from the system, leaving voids behind. Primarily, the reason for using He^+ ion irradiation is to avoid implantation, and encourage defect-formation via knock-on damage. He bubbles can sometimes get trapped in polycrystalline materials[5]. However, not surprisingly we see no evidence of He bubble trapping from our TEM images. In Supplementary Figure 4, we show some more TEM images of our

nanowires which clearly demonstrate the non-existence of these bubbles. A general procedure to observe the voids, which are left from He bubbles escaping, is via defocused bright field TEM images[6]. However in our single-crystalline nanowires we do not observe these voids too, and this clearly suggests that there is no He^+ implantation which subsequently either creates bubbles or voids (see also Supplementary Note 1).

Supplementary Note 6: Profiles of applied voltage pulses and current response

Shapes of the applied voltage pulses and their current response through the devices are measured through a 500 MHz oscilloscope (measurement procedure is described in Methods). Supplementary Figure 5 shows that for a 50 ns applied voltage pulse, the current is mostly at a steady value during the pulse, given by the ratio of applied voltage (V) and the resistance measured after removing the pulse (R). Hence current in the device can be approximated as a rectangular pulse of amplitude V/R. For a 20 ns voltage pulse, however, the reflections corresponding to slight changes in the voltage pulse dominate, and the rectangular current pulse assumption is no longer a valid one. Our discussion in the manuscript, about formation of intermediate states starting from state 1 by applying 20 ns voltage pulses, hence did not contain any quantification of the current values.

Supplementary Note 7: Nanowire switching behavior at dosages where carrier localization effects are not significant (less than 700 μC cm^{-2})

As shown in Supplementary Figure 6a, at dosages up to 700 μC cm^{-2} where carrier localization effects are not significant, we observe the following trends in switching behavior: (i) Switching current density (j_s) increases with increasing l_d up to a critical length, l_c, demonstrating volume scaling of switching current up to l_c, (ii) j_s suddenly drops at l_c and subsequently increases again with length, and (iii) l_c itself decreases with increasing dosage.

7.1. Size scaling of switching currents up to l_c

In the defect-templated pathway for amorphization, heat shock from a current pulse is responsible for quenching of vacancy clusters into extended defects[4,7], which migrate with the hole-wind force, and keep piling up at a region of local inhomogeneity up until a local collapse of long-range order (amorphization). The switching currents in this pathway scale with device volumes, and this can be understood by separately considering the effect of a current pulse on devices of same cross sectional area and varying lengths; and devices of varying cross-sectional areas and same length.

7.1.1. Comparison of the effect of a current pulse on nanowire devices of different lengths and same cross-sectional area:

We performed finite element simulations of temperature profiles in a nanowire device upon the application of a current pulse, using COMSOL[8], to understand the role of heat-shocks in the length scaling behavior of switching currents for devices switched via the defect-templated pathway. The geometry of the GeTe nanowires (thermal conductivity, κ=0.5 W per mK; thermal diffusivity, α=5x10^{-3} cm^2 sec^{-1}; and electrical resistivity ρ=0.4 mΩ cm) was

simulated as long bars with square cross-section– the diameter of the nanowire being the width and height of the bar. The ends of the nanowire devices were considered to be the heat sinks (electrode regions), and the entire device was embedded in an SiO_x dielectric of thickness 30 nm (κ=1.6 W per mK, α=0.1 cm^2 sec^{-1}). Current amplitudes were set to the desired values and pulses were defined as rectangular functions of current with rising and trailing edges of width 2.5 ns each. Spatial profile, 120 ns after the application of 100 ns, 0.4 mA pulse (120 ns is the time instant when maximum temperature is reached) on a 2500 nm x 100 nm x 100 nm nanowire device is shown in Supplementary Figure 6b.

To compare the effect of a current pulse (0.4 mA, 100 ns) on devices of different lengths and same cross-sectional area, we calculated the temporal profile of the temperature at the mid-point of several devices (which is spatially the maximum temperature region at every time instant) with varying lengths (900-2700 nm). We find that with increasing the length of the device, quench times for the heat shocks corresponding to a particular current pulse become longer (Supplementary Figure 6c), making their severity lesser and hence the defect creation and migration process less effective. Hence, under a valid assumption that the defect density required for amorphization is size independent, longer devices require higher currents to achieve this critical density than shorter devices, and this explains the length dependence of switching currents.

7.1.2 Comparison of the effect of a current pulse on nanowire devices of different cross-sectional areas and the same length:

It is trivial to understand that any particular current pulse will heat a thinner device to a higher temperature than a thicker device of the same length, as the current density is lesser in a thicker device. Defect creation (and subsequent migration) from a current pulse in a thinner device is hence more effective than that in a thicker device, manifesting as higher switching currents for thicker devices, thus explaining scaling of switching currents with cross-sectional area.

Combining both the length scaling as well as cross-sectional area scaling of switching currents, we can conclude that the switching currents scale with the volume of the device in defect-templated amorphization pathway also (just as in melt-quench pathway).

7.2 Sudden drop of switching current density at l_c

The pre-induced defects and the defects created by the heat shock during electrical pulsing, migrate with the electric wind force and accumulate at a region of local inhomogeneity– defined by structural, morphological or thermal factors– which impedes the motion of defects[4,7]. Beyond a critical concentration of defect pile-up in this local region, an amorphous phase nucleates[7], and it is easy to argue statistically that longer devices have more of such inhomogeneities than the shorter ones. Hence, longer devices may be treated as many short segments, each containing a defect-templating location. Defect build up towards amorphization at these templates in all the segments happens simultaneously, with the shortest segment determining the ease of switching. Devices just longer than l_c have one extra defect-templating location and hence an extra shorter segment than the devices just shorter than l_c. This explains the sudden drop of switching current as a function of device length at l_c at all the dosages until 700 μC cm^{-2}. Furthermore, since pre-induced defects act as natural inhomogeneities, it is easy to create multiple jamming locations in devices irradiated at higher dosages (greater concentration

of pre-induced defects); and this is reflected as decrease in l_c with increasing dosage (Supplementary Figure 6a).

7.3. Switching currents increase with dosage at lower dosages (less than, 700 μC cm^{-2})

From a structural point of view, in defect-templated amorphization pathway if extended defects are pre-induced, energy expense for both creation and migration of the defects, can be massively reduced, and this translates to reduction in switching currents (and current densities). Contrarily, we note from the data in Supplementary Figure 6a that the switching currents increased with increasing dosage up to 700 μC cm^{-2} (where defects do not induce any carrier localization effects in transport). This behavior can be understood by noting the trends in programming curve (device steady state resistance as a function of voltage pulse amplitude) of the devices that were ion-irradiated, and those that did not (Supplementary Figure 6d). The ion-irradiated devices at low dosages (less than 700 μC cm^{-2}) show a clear dip in the resistance as a function of voltage pulse amplitude –not prominent in non-irradiated devices. The dip in resistance from the initial value is a result of pre-induced defect annealing or reorganization in a manner that increases the carrier mobility by reducing carrier-defect scattering. This defect reorganization requires more work to be done on the system (than the case with no pre-induced defects), which manifests itself as higher switching currents. Hence, low-concentration of pre-induced defects which electronically do not modify the material is bad in terms of energy consumption for the crystal-amorphous switching process.

Supplementary Note 8: RESET and SET behavior of devices defect-engineered into electronic states in crystalline phase where localized carriers participate in transport

8.1. Determination of active volumes and active areas:

From previous works on defect-templated amorphization in both GeTe[4] and $Ge_2Sb_2Te_5$[7] we know that amorphous region forms in a local region cutting across the entire cross-section of the nanowire, thus making the active area as the total cross sectional area of the device. The defect evolution responsible for amorphization happens throughout the nanowire device (between two electrodes), and thus the active volume is the total device volume.

8.2. Threshold switching via d.c. I-V sweep:

In Supplementary Figure 7, we show threshold switching data to SET state of device D2 (320 nm x 80 nm x 80 nm) exposed to 3600 μC cm^{-2}, which amorphized at a very low RESET current of 8 μA (0.13 MA cm^{-2}; see Fig. 4 of the manuscript).

Supplementary Note 9: Intermediate states, visualization and stability:

9.1 Visualization of intermediate states:

The intermediate states are metastable crystalline states, which microstructurally can be described as having a background concentration of defects in most of the nanowire, and a local region where the defect concentration exceeds the background. Supplementary Figure 8 shows TEM images of two of our devices, which were programmed to one of the intermediate states. However, it must be noted that various intermediate states structurally differ by concentration difference in the defects in the defect-template region. Small changes in defect density in the

defect-template are difficult to characterize using diffraction contrast TEM, and hence we rely on transport measurements to deduce both structural and electronic information about these states.

9.2 Thermal stability of intermediate states and the amorphous phase

We examined the thermal stability of the amorphous phase and an intermediate state –a limiting factor that determines the data non-volatility, by performing high temperature retention measurements, as practical memory applications warrant high-temperature performance[9]. Isothermal crystallization from an amorphous phase or an intermediate state at high temperatures show an initial incubation regime (no change in resistance) corresponding to the time required for the formation of a critical nucleus of the crystal, followed by a growth regime[9] (Supplementary Figure 9a, b). We considered data retention times as the incubation times, and in both these states (amorphous and intermediate) the incubation time-temperature plots show an Arrhenius behavior (t=Pexp(E_a/kT), Supplementary Figure 9c)[9,11] , with the amorphous phase displaying excellent thermal stability, extrapolated to 3.1 years for device operations at 115°C. The intermediate state is however, not very stable for high temperature operations i.e. for operations at 40°C, extrapolated stability is 3.1 years, whereas for operations at 70°C it is barely 20 minutes; and improving the thermal stability of these intermediate electronic states is an interesting problem for future work.

9.3 Obtaining intermediate states starting from defect-engineered insulating crystalline state

In Supplementary Figure 10 we show additional data to Fig. 4a on another device (D3, which also amorphizes abruptly at 23 μA with the application of 50 ns pulses, but which proceeds through intermediate states by controlled addition of defects upon the application of 20 ns pulses.

Supplementary Note 10: Model for recrystallization

After amorphization, the nanowires have the following microstructure: most of the nanowire has a background density of pre-induced defects, and a local region(s) has higher defect concentration (defect-template). From previous works on defect-templated amorphization[4,7] we know that the amorphous region cuts across the cross-section of the nanowire in this template wherever the defect concentration exceeded a critical value required for amorphization (Supplementary Figure 11a, b). So in this local region of the defect-template, there is an amorphous region surrounded by a heavily defective crystalline region with the defect concentration exceeding the background concentration (Supplementary Figure 11). The reverse process, the clues for which are obtained from this work for the first time, involves two steps: a) threshold switching followed by recrystallization of the amorphous region, just as it happens in the melt-quench strategy and b) reduction of defect concentration in the rest of the template through homogenization of defects via Joule heating (Supplementary Figure 11c shows recrystallized device, with almost uniform defect contrast suggesting defect-homogenization). The latter process, i.e. degree of defect homogenization, provides access to

several metastable intermediate resistance states in crystalline phase (see Supplementary Note 11).

Supplementary Note 11: Threshold switching and amorphization of intermediate states using voltage sweeps

Amorphization of intermediate states using d.c. IV sweeps clearly demonstrates that the defect-templated pathway is a purely solid-state amorphization pathway (Supplementary Figure 12a). Previous works on defect-templated amorphization mechanism[4,7] remained agnostic about the exact nature of amorphization after the formation of entangled defect-template. While adding more disorder through hole-wind force to the already heavily disordered defect-templated region, can collapse the local structure to an amorphous state without invoking the liquid phase; there is also a possibility that defect-template could tremendously reduce the local melting point, with the heat in the voltage pulse sufficient to melt and quench this templated region. Here, by being able to amorphize the metastable defect-templated structure through very low d.c. currents (approximately, 0.1 μA , and not a pulse), we removed the possibility of quenching, convincingly showing that it is just the hole-wind force that is responsible for defect accumulation, and the crystal-amorphous transformation is completely solid-state.

Supplementary Figure 12b schematically summarizes the I-V behavior of the intermediate states (structurally signified as crystalline nanowires with heavy local disorder forming defect template): the hole-wind force corresponding to very low currents, is sufficient to transform the jammed region through more defect accumulation, into an amorphous phase which shows threshold switching behavior. Depending on how the Joule heating that follows threshold switching is controlled, the system may transform reliably into intermediate states (states 2 and 3), or the recrystallized state resembling the as-engineered insulating state (state 1).

Supplementary Note 12: Physical understanding of the amorphous phase formed via defect-templated approach

The amorphous phase characteristics found in this work are different from the amorphous phase formed from melt-quench pathway. From Fig. 6b (temperature-conductance measurements on the amorphous phase) we observe that in the high temperature range (160-300 K), there is no thermal activation of the carriers into the delocalized states, rather they still follow the hopping conduction. Based on the limited evidence we have, we speculate the following hypothesis based on ref. 12 to explain the observed transport characteristics:

Atomic arrangement in the amorphous phase can be described as continuous random network with ‘defects’, which electronically generate carrier trap states (donor, acceptor or deep traps) in the mobility gap. Typically, every trap state contains one electron, and the addition of another electron to this state will add a Coulomb energy penalty to the system. In chalcogenide semiconductors, these one-electron centers, if present, are neutral. However in chalcogenide glasses, it is known that the concentration of these one-electron centers or neutral traps (D^o) is much less compared to the charged traps, which contain either 2 electrons or 2 holes (D^+, D^-). These charged traps are known as negative U centers, and the Coulomb energy penalty owing to two carriers trapped in the same state is counter balanced by the large stabilization energy owing to the bi-polaron formation (two trapped electrons + lattice distortion) with the help of the lattice. The bi-polarons by themselves do not participate in DC conduction. So the

conduction pathway for an injected carrier in these glasses is either by getting activated to delocalized states, or by hopping between the sparsely populated one electron centers. The formation of bi-polarons (donor and acceptor) pins the Fermi level to the mid-gap, and also increases the activation energy for conduction into delocalized states, than the value of $E_{gap}/2$ (typically observed for intrinsic semiconductors). In regular chalcogenide glasses, at low temperatures where carrier activation is difficult owing to the enhanced activation energy due to Coulomb repulsion, hopping conduction dominates and is the only possible way of conduction despite the low-concentration of single-electron traps. At high temperatures, however, energy is sufficient for activated conduction to dominate at the cost of hopping conduction.

The observations on our amorphous phase (no activation to delocalized states, and VRH at high temperatures), which is formed by accumulating extended defects in crystalline phase, on the other hand, suggest there are a lot more neutral trap centers (D^o) as compared to the melt-quench amorphous phase. Hopping probability depends on the distance between the traps both spatially and energetically. By increasing the number of these trap states, the trap to trap distance is reduced (both in energy and in space), enhancing the chances of hopping as against the chances of activation into delocalized states at higher temperatures. Our temperature-conductance measurements, discussed as a response to the next comment, support this hypothesis.

The low-temperature data can have many fits, one possibility being $T^{-0.5}$ (another being $T^{-0.25}$) as we show in Supplementary Figure 13, and hence we did not show any particular fit in Fig. 6b. The $T^{-0.5}$ fit can be consistent with the hypothesis of existence of all the three traps: positive, negative and neutral centers. It is known in systems with negative U centers (charged traps in chalcogenide semiconductors) that a Coulomb gap opens up among these defect states, modifying the low-temperature transport to an Efros-Shklovskii (E-S) hopping conduction (described by a $T^{-0.5}$ dependence of log S). This crossover behavior from VRH to E-S with reduction in temperature is well studied in various materials systems[13]. However, such a hypothesis can only be conclusively shown by techniques such as electron spin resonance (e.s.r.), and very low-temperature conductivity measurements, which is a subject of future research.

Supplementary References:

1. Park, M.-A., Savran, K., Kim, Y-J. Weak localization and the Mooij rule in disordered materials, *phys. stat. sol.* **237**, 500-512 (2003).

2. Raether, H. *Excitation of plasmons and interband transitions by electrons*. Springer tracts in modern physics, Vol. 88 (Springer-Verlag: New York, 1980).

3. Edwards, A. H. et al., Electronic structure of intrinsic defects in crystalline germanium telluride. *Phys. Rev. B* **73**, 045210 (2006).

4. Nukala, P. *et al.* Direct observation of metal-insulator transition in single-crystalline germanium telluride nanowire memory devices prior to amorphization. *Nano Lett.* **14**, 2201-2209 (2014)

5. Jenkins, M.L., Kirk, M.A., *Characterization of radiation damage by transmission electron microscopy*, Taylor & Francis (2000).

6. Abrams, K.J., et al., Helium irradiation effects in polycrystalline Si, silica, and single crystal Si. *J. Appl.Phys*. **111**, 083527 (2012).

7. Nam, S. W. *et al.* Electrical wind force-driven and dislocation-templated amorphization in phase-change nanowires. *Science* **336**, 1561-1566 (2012).

8. Multiphysics and simulation software: COMSOL. http://www.comsol.com/

9. Lee, S-H., Jung. Y., Agarwal. R., Highly scalable non-volatile and ultra-low-power phase-change nanowire memory. *Nature Nanotech.* **2**, 626-630 (2007).

10. Senkader, S., Wright, C. D. Models for phase-change of $Ge_2Sb_2Te_5$ in optical and electrical memory devices, *J.Appl.Phys.* **95,** 504-511 (2004).

11. Weidenhof, V., Friedrich, I., Ziegler, S., Wuttig, M., Laser induced crystallization of amorphous $Ge_2Sb_2Te_5$ films, *J.Appl.Phys.* **89,** 3168-3176 (2001).

12. Mott, N.F., Davies, E.A., *Electronic processes in non-crystalline materials*, Vol.2 (Oxford University Press Inc, 1979)

13. Rozenbaum. R., Crossover from Mott to Efros-Shklovskii variable-range-hopping conductivity in In_xO_y films, *Phys. Rev. B* **44**, 3599-3603 (1991).
